# Supplementary material for: Average nucleotide identity-based Staphylococcus aureus strain grouping allows identification of strain-specific genes in the pangenome
Source: mSystems. 2024 Jun 27;9(7):e00143-24. doi: 10.1128/msystems.00143-24 (PMC11265343; doi:10.1128/msystems.00143-24)
Supplement: Supplemental figures and table — Figures S1 to S8 and Table S1. [file msystems.00143-24-s0001.docx]

- **Supplemental Data**

**Fig S1**

**Figure S1 legend**

Figure S1: Effect of filtering, clustering and dereplicating 83,383 *S. aureus* genomes

(**A**) The x-axis shows the total number of variants when compared with the Bactopia auto-chosen reference, and the y-axis shows the average minor allele frequency (MAF). Each dot is one of 57,093 genomes which were obtained after filtering out samples ranked ‘Bronze’ or ‘Exclude’ by Bactopia and/or found to have non-*S. aureus* genome content by Bactopia and CheckM (Figure 1). Samples in the top quadrant (Above red horizontal line - Average MAF > 0.05) were considered to be *S. aureus* strain mixtures and were discarded. The remaining 56,771 samples in the bottom quadrant (< 0.05 Average MAF) were used for further analysis. (**B**) Boxplots showing spread of pairwise ANI within each “strain” cluster. Only strain clusters having more than 10 genomes are shown. Black horizontal line within each boxplot shows the median within strain-cluster pairwise ANI.

Total number of unique genes discovered (**C**) and total number of strains discovered (**D**) for every new genome added from the dereplicated set (red dots) or a random genome from the un-dereplicated 58,034 (blue dots). Up to 1000 random genomes were added from each set and the total number of unique genes or strains were measured for every genome added (light red and light blue dots). This procedure was repeated 5 times and the median number of genes or strains discovered are shown in dark red and dark blue dots. More genes and more strains were discovered from the same number of genomes (after observing 1000 genomes) in the dereplicated set compared to the un-dereplicated set.

**Figure S2**

**Figure S2 Legend**

Figure S2: The 7,954 substrain pangenome of *S. aureus*.

Histograms with 30 bins each, depicting the (**A**) frequency distribution of genes in our dataset, (**B**) the average dosage of each gene per genome, (**C**) the average length distribution of each gene, and (**D**) the distribution of the number of unique PIRATE gene families per genome.

**Figure S3**

**Figure S3 legend**

Dot plot showing percentage prevalence of only intermediate genes (> 10%, < 95%) on the x-axis and the corresponding F_ST_ on the y-axis. F_ST_ scores calculated for (**A**) *agr* type-based population segregation in the 7954 set and (**B**) Strain-based population segregation in the 740 set.

**Figure S4**

**Figure S4 legend**

Figure S4: strain-concentrated gene content declines gradually with core-gene distance.

Each dot represents a comparison between substrains in the 740-set. Patristic distance was tip-tip distance on the phylogeny. Hamming distance was calculated from a presence absence matrix of each non-core gene type: (**A**) rare genes, (**B**) strain-diffuse, (**C**) strain-concentrated, (**D**) all non-core (note different y-axis scale). Red lines show the linear model fit.

**Figure S5**

**Figure S5 legend**

Figure S5: Genomad score distributions for 7954-set pangenomes.

The GeNomad [(27)](https://paperpile.com/c/sbIlO5/qDMOU) probability scores for (**A**) chromosome (**B**) plasmid and (**C**) virus were grouped by gene class (Core, Strain-concentrated, Strain-diffuse, rare). A score of 1 indicates maximum confidence that the given gene is chromosome/plasmid/virus associated according to GeNomad. All differences were significant in a Tukey's pairwise comparisons at < 0.05 (corrected for multiple tests), except strain-diffuse-Core plasmid score and strain-concentrated-Core virus score. Each box represent the 25-75 interquartile range and the line shows the 50th percentile.

**Figure S6**

**Figure S6 legend**

Figure S6 Relationship between gene prevalence, number of strains and homoplasy for non-core genes for the 7954-set (a,b) and the 740-set-90 (c,d)

The plots are formatted as Figure 5. Each dot represents a non-core gene. “concentrated” = strain-concentrated, “diffuse” = strain-diffuse. Panels A and C show the relationship between overall prevalence (number of genomes out of 740) and number of strains (out of 37) each gene is found in. Panels B and D show the relationship between prevalence of estimated number of changes on the species tree calculated by homoplasyfinder[(26)](https://paperpile.com/c/sbIlO5/gyMUh). In panel A, the unbalanced nature of the 7954-set (a few strains have thousands of genomes, many have only one) obscures the differences between concentrated and diffuse: it not possible to plot simple bounds of lowest possible and random gene distribution into strains as it is for the 740-90 set (panel C).

**Figure S7**

**Figure S7 Legend**

Figure S7: Chromosome start locations of non-core genes on six *S.aureus* complete chromosomes.

The name on the left-hand side refers to NCBI assembly database designations. GCA_014731755 is CC30 MRSA; GCA_000160335 is CC30 MSSA; GCA_000024585 is CC5 MSSA; GCA_0000134525 is CC8 MRSA; GCA_000012045 is CC8 MRSA; GCA_000009645 is CC5 MRSA (N315 the *S. aureus* type strain). “Intermediate_Hi_Fst” = strain-concentrated; “Intermediate_Lo_Fst” = strain-diffuse.

**Figure S8**

**Figure S8 legend**

Figure S8: Average nucleotide identity (ANI) vs core gene SNP distance

All vs. all pairwise ANI and SNP distances from core gene concatenations are plotted on the y and x axis respectively. The black line represents a linear model and the red horizontal line is drawn at ANI 99.5%. The red vertical line is drawn at the intersection between 99.5% ANI and the linear model.

Table S1: *S. aureus* studies quoting pangenome statistics.

**“?” indicates that the corresponding information could not be found**

| **Title** | **Date** | **No. of genomes** | **Sampling space** | **Assembly level** | **Pangenome tool** | **No. core** | **Total gene families** |
| --- | --- | --- | --- | --- | --- | --- | --- |
| Comparative Pan-Genomic Analysis Revealed an Improved Multi-Locus Sequence Typing Scheme for Staphylococcus aureus [(1)](https://paperpile.com/c/HyI9fO/PnMeW) | 2022-11-19 | 502 | Diverse | Complete | PanRV (Roary) | 2320 | 12477 |
| Pan-Genome Analysis of Staphylococcus aureus Reveals Key Factors Influencing Genomic Plasticity [(2)](https://paperpile.com/c/HyI9fO/fCGuK) | 2022-11-01 | 1519 | Diverse | All | Roary | 1000 | 16794 |
| Pangenomic Approach To Understanding Microbial Adaptations within a Model Built Environment, the International Space Station, Relative to Human Hosts and Soil [(3)](https://paperpile.com/c/HyI9fO/vOYuP) | 2022-01-08 | 106 | ISS, human, soil | All | Roary | 1935 | 6847 |
| The Epidemiological and Pangenome Landscape of Staphylococcus aureus and Identification of Conserved Novel Candidate Vaccine Antigens [(4)](https://paperpile.com/c/HyI9fO/EMWij) | 2022-02-01 | 355 | Diverse | All | ? | 2025 | 7199 |
| Analyses of Livestock-Associated Staphylococcus aureus Pan-Genomes Suggest Virulence Is Not Primary Interest in Evolution of Its Genome [(5)](https://paperpile.com/c/HyI9fO/Wuxe3) | 2019-05-22 | 14 | Livestock associated | Complete | Roary | 1969 | 4637 |
| Comparative genome-scale modelling of Staphylococcus aureus strains identifies strain-specific metabolic capabilities linked to pathogenicity [(6)](https://paperpile.com/c/HyI9fO/dxoq8) | 2016-06-10 | 64 | Diverse | All | dGenome DuctAPE | 1441 | 7457 |
| PanRV: Pangenome-reverse vaccinology approach for identifications of potential vaccine candidates in microbial pangenome [(7)](https://paperpile.com/c/HyI9fO/z9Q9u) | 2019-03-12 | 301 | Diverse | All | PanRV (Roary) | 1524 | 11384 |
| Whole-Genome Sequencing of Staphylococcus aureus and Staphylococcus haemolyticus Clinical Isolates from Egypt [(8)](https://paperpile.com/c/HyI9fO/Lg1uM) | 2022-06-21 | 90 | 56 from 1 hospital and 34 from greater Arab region | All | Anvio | 1501 | 4283 |
| Phylogenomic Analysis Reveals the Evolutionary Route of Resistant Genes in Staphylococcus aureus [(9)](https://paperpile.com/c/HyI9fO/XKtmH) | 2019-11-03 | 152 | Diverse | Complete | Manual alignment and clustering | 2426 | 6326 |
| Comparative genomic analysis of Staphylococcus aureus isolates associated with either bovine intramammary infections or human infections demonstrates the importance of restriction-modification systems in host adaptation [(10)](https://paperpile.com/c/HyI9fO/PV8g6) | 2022-02-18 | 187 | Human and cattle | All | Roary | 2700 | 6812 |
| Molecular Epidemiology of Staphylococcus aureus in China Reveals the Key Gene Features Involved in Epidemic Transmission and Adaptive Evolution [(11)](https://paperpile.com/c/HyI9fO/B7rT0) | 2022-10-03 | 332 | Human clinical strains from China | All | Heap's law algorithms | 890 | 5832 |
| Estimated Roles of the Carrier and the Bacterial Strain When Methicillin-Resistant Staphylococcus aureus Decolonization Fails: a Case-Control Study [(12)](https://paperpile.com/c/HyI9fO/JJkKv) | 2022-08-24 | 477 | MRSA carriers from Denmark hospitals | All | panX | 1671 | 5925 |
| Forecasting Staphylococcus aureus Infections Using Genome-Wide Association Studies, Machine Learning, and Transcriptomic Approaches [(13)](https://paperpile.com/c/HyI9fO/Zkop7) | 2022-07-05 | 356 | Mostly human | All | Panaroo | 1489 | 8827 |
| Carriage prevalence and genomic epidemiology of Staphylococcus aureus among Native American children and adults in the Southwestern USA [(14)](https://paperpile.com/c/HyI9fO/qeqiX) | 2022-05-13 | 92 | Native Americans from Southwestern USA | Complete | Roary | 1808 | ? |
| Polyclonality, Shared Strains, and Convergent Evolution in Chronic Cystic Fibrosis Staphylococcus aureus Airway Infection [(15)](https://paperpile.com/c/HyI9fO/UJgI4) | 2020-03-23 | 1382 | Longitudinal sampling from 246 children with CF from the US | All | Roary | 1142 | 21358 |
| PIRATE: A fast and scalable pangenomics toolbox for clustering diverged orthologues in bacteria [(16)](https://paperpile.com/c/HyI9fO/nplVA) | 2019-10-09 | 253 | Diverse | All | PIRATE | 2433 | 4250 |
| Whole-Genome Sequencing for Routine Pathogen Surveillance in Public Health: a Population Snapshot of Invasive Staphylococcus aureus in Europe [(17)](https://paperpile.com/c/HyI9fO/1SsUd) | 2016-05-05 | 308 | Invasive isolates from Europe hospitals within a 6 month period | All | BlastP & TribeMCL | ? | 4281 |

1. [Jalil M, Quddos F, Anwer F, Nasir S, Rahman A, Alharbi M, Alshammari A, Alshammari HK, Ali A. 2022. Comparative Pan-Genomic Analysis Revealed an Improved Multi-Locus Sequence Typing Scheme for Staphylococcus aureus. Genes 13.](http://paperpile.com/b/HyI9fO/PnMeW)

2. [Liu N, Liu D, Li K, Hu S, He Z. 2022. Pan-Genome Analysis of Staphylococcus aureus Reveals Key Factors Influencing Genomic Plasticity. Microbiol Spectr 10:e0311722.](http://paperpile.com/b/HyI9fO/fCGuK)

3. [Blaustein RA, McFarland AG, Ben Maamar S, Lopez A, Castro-Wallace S, Hartmann EM. 2019. Pangenomic Approach To Understanding Microbial Adaptations within a Model Built Environment, the International Space Station, Relative to Human Hosts and Soil. mSystems 4:e00281–18.](http://paperpile.com/b/HyI9fO/vOYuP)

4. [Naz K, Ullah N, Naz A, Irum S, Dar HA, Zaheer T, Shahid F, Ali A. 2022. The Epidemiological and Pangenome Landscape of Staphylococcus aureus and Identification of Conserved Novel Candidate Vaccine Antigens. Curr Proteomics 19:114–126.](http://paperpile.com/b/HyI9fO/EMWij)

5. [Rao RT, Sivakumar N, Jayakumar K. 2019. Analyses of Livestock-Associated Staphylococcus aureus Pan-Genomes Suggest Virulence Is Not Primary Interest in Evolution of Its Genome. OMICS 23:224–236.](http://paperpile.com/b/HyI9fO/Wuxe3)

6. [Bosi E, Monk JM, Aziz RK, Fondi M, Nizet V, Palsson BØ. 2016. Comparative genome-scale modelling of Staphylococcus aureus strains identifies strain-specific metabolic capabilities linked to pathogenicity. Proc Natl Acad Sci U S A https://doi.org/HYPERLINK "http://dx.doi.org/10.1073/pnas.1523199113"10.1073/pnas.1523199113HYPERLINK "http://paperpile.com/b/HyI9fO/dxoq8".](http://paperpile.com/b/HyI9fO/dxoq8)

7. [Naz K, Naz A, Ashraf ST, Rizwan M, Ahmad J, Baumbach J, Ali A. 2019. PanRV: Pangenome-reverse vaccinology approach for identifications of potential vaccine candidates in microbial pangenome. BMC Bioinformatics 20:123.](http://paperpile.com/b/HyI9fO/z9Q9u)

8. [Montelongo C, Mores CR, Putonti C, Wolfe AJ, Abouelfetouh A. 2022. Whole-Genome Sequencing of Staphylococcus aureus and Staphylococcus haemolyticus Clinical Isolates from Egypt. Microbiol Spectr 10:e0241321.](http://paperpile.com/b/HyI9fO/Lg1uM)

9. [John J, George S, Nori SRC, Nelson-Sathi S. 2019. Evolutionary route of resistant genes in Staphylococcus aureus. Genome Biol Evol https://doi.org/HYPERLINK "http://dx.doi.org/10.1093/gbe/evz213"10.1093/gbe/evz213HYPERLINK "http://paperpile.com/b/HyI9fO/XKtmH".](http://paperpile.com/b/HyI9fO/XKtmH)

10. [Park S, Jung D, O’Brien B, Ruffini J, Dussault F, Dube-Duquette A, Demontier É, Lucier J-F, Malouin F, Dufour S, Ronholm J. 2022. Comparative genomic analysis of Staphylococcus aureus isolates associated with either bovine intramammary infections or human infections demonstrates the importance of restriction-modification systems in host adaptation. Microb Genom 8.](http://paperpile.com/b/HyI9fO/PV8g6)

11. [Xu Z, Yuan C. 2022. Molecular Epidemiology of Staphylococcus aureus in China Reveals the Key Gene Features Involved in Epidemic Transmission and Adaptive Evolution. Microbiol Spectr e0156422.](http://paperpile.com/b/HyI9fO/B7rT0)

12. [Holm MKA, Jørgensen KM, Bagge K, Worning P, Pedersen M, Westh H, Monk JM, Bartels MD. 2022. Estimated Roles of the Carrier and the Bacterial Strain When Methicillin-Resistant Staphylococcus aureus Decolonization Fails: a Case-Control Study. Microbiol Spectr e0129622.](http://paperpile.com/b/HyI9fO/JJkKv)

13. [Sassi M, Bronsard J, Pascreau G, Emily M, Donnio P-Y, Revest M, Felden B, Wirth T, Augagneur Y. 2022. Forecasting Staphylococcus aureus Infections Using Genome-Wide Association Studies, Machine Learning, and Transcriptomic Approaches. mSystems e0037822.](http://paperpile.com/b/HyI9fO/Zkop7)

14. [Cella E, Sutcliffe CG, Tso C, Paul E, Ritchie N, Colelay J, Denny E, Grant LR, Weatherholtz RC, Hammitt LL, Azarian T. 2022. Carriage prevalence and genomic epidemiology of Staphylococcus aureus among Native American children and adults in the Southwestern USA. Microbial Genomics 8:000806.](http://paperpile.com/b/HyI9fO/qeqiX)

15. [Long DR, Wolter DJ, Lee M, Precit M, McLean K, Holmes E, Penewit K, Waalkes A, Hoffman LR, Salipante SJ. 2020. Polyclonality, Shared Strains, and Convergent Evolution in Chronic CF S. aureus Airway Infection. Am J Respir Crit Care Med https://doi.org/HYPERLINK "http://dx.doi.org/10.1164/rccm.202003-0735OC"10.1164/rccm.202003-0735OCHYPERLINK "http://paperpile.com/b/HyI9fO/UJgI4".](http://paperpile.com/b/HyI9fO/UJgI4)

16. [Bayliss SC, Thorpe HA, Coyle NM, Sheppard SK, Feil EJ. 2019. PIRATE: A fast and scalable pangenomics toolbox for clustering diverged orthologues in bacteria. Gigascience 8:598391.](http://paperpile.com/b/HyI9fO/nplVA)

17. [Aanensen DM, Feil EJ, Holden MTG, Dordel J, Yeats CA, Fedosejev A, Goater R, Castillo-Ramírez S, Corander J, Colijn C, Chlebowicz MA, Schouls L, Heck M, Pluister G, Ruimy R, Kahlmeter G, Åhman J, Matuschek E, Friedrich AW, Parkhill J, Bentley SD, Spratt BG, Grundmann H, European SRL Working Group. 2016. Whole-Genome Sequencing for Routine Pathogen Surveillance in Public Health: a Population Snapshot of Invasive Staphylococcus aureus in Europe. MBio 7.](http://paperpile.com/b/HyI9fO/1SsUd)
